# Emerging Role of Corticosteroid-Binding Globulin in Glucocorticoid-Driven Metabolic Disorders

**DOI:** 10.3389/fendo.2016.00160

**Published:** 2016-12-19

**Authors:** Marie-Pierre Moisan, Nathalie Castanon

**Affiliations:** ^1^INRA, Nutrition and Integrative Neurobiology (NutrINeurO), UMR 1286, Bordeaux, France; ^2^Université de Bordeaux, Nutrition and Integrative Neurobiology (NutrINeurO), UMR 1286, Bordeaux, France

**Keywords:** glucocorticoids, transcortin, obesity, metabolism, lipid storage

## Abstract

Glucocorticoid hormones (GCs) are critical for survival since they ensure the energy supply necessary to the body in an ever challenging environment. GCs are known to act on appetite, glucose metabolism, fatty acid metabolism, and storage. However, to be beneficial to the body, GC levels should be maintained in an optimal window of concentrations. Not surprisingly, conditions of GC excess or deficiency, e.g., Cushing’s syndrome or Addison’s disease, are associated with severe alterations of energy metabolism. Corticosteroid-binding globulin (CBG), through its high specific affinity for GCs, plays a critical role in regulating plasma GC levels and their access to target cells. Genetic studies in various species including humans have revealed that CBG is the major factor influencing interindividual genetic variability of plasma GC levels, both in basal and stress conditions. Some, but not all, of these genetic studies have also provided data linking CBG levels to body composition and insulin levels. The examination of CBG-deficient mice submitted to hyperlipidic diets unveiled specific roles for CBG in lipid storage and metabolism. An influence of CBG on appetite has not been reported but remains to be more finely analyzed. Finally, only male mice have been examined under high-fat diet, while obesity is affecting women even more than men. Overall, a role of CBG in GC-driven metabolic disorders is emerging in recent studies. Although subtle, the influence of CBG in these diseases could open the way to new therapeutic interventions since CBG is easily accessible in the blood.

## Introduction

Glucocorticoid hormones (GCs) are adrenal cortex steroids that were named from their role in the regulation of glucose metabolism, mostly through maintaining the reserves of glycogen in the liver. They also play an intricate role in fatty acid metabolism and storage. Finally, they are also important regulators of appetite through complex interactions with orexigenic and anorexigenic neuropeptides and hormones. Excess GC levels, encountered in Cushing’s syndrome or GC therapy, lead to central obesity associated with hyperphagia, insulin resistance, and fatty liver development (1).

Under normal physiological conditions, GC secretion (cortisol or corticosterone depending on species) follows a circadian rhythm entrained by light and food intake. These environmental stimuli trigger the secretion of the hypothalamic peptide corticotrophin-releasing hormone (CRH), which, in turn, stimulates pituitary secretion of adrenocorticotrophic hormone (ACTH) that acts on adrenal glands to induce GC synthesis and release in the blood (2). In plasma, GCs bind with a high affinity but low capacity to transcortin, also called corticosteroid-binding globulin (CBG), and to albumin with a high capacity but low affinity (3, 4). The free fraction of circulating GCs thus constitutes around 3–5% of the total GC pool. Free GCs regulate negatively their own secretion by inhibiting CRH and ACTH release and act on target tissues by binding to specific receptors, the mineralocorticoid and the GC receptors (5). Under stressful conditions, the increase in CRH levels results in increased GC secretion that must be transient to be protective to the body. GCs are essential for survival but not enough or too much GC signaling is deleterious for health (6).

Corticosteroid-binding globulin has been known since 1956 as a plasma protein capable of high-affinity interaction with cortisol (4). It was purified in the early 60s and cloned and characterized since the 90s in many species (7). Recently, CBG has been identified as a major component of GC genetic variability in animals and humans. Consequently, the role of CBG in GC-driven metabolic disorders and obesity gained interest. This mini-review summarizes the recent studies related to the role of CBG in GC-driven metabolic alterations and obesity.

## Genetic Studies Highlighting the Central Role of CBG in GC Variability

Large individual differences exist in both basal and stress-induced GC levels. Because this variability is in part of genetic origin, several research groups have used genetic mapping on experimental crosses, i.e., a non-hypothesis-driven approaches, to identify genetic factors contributing to this variability of GC levels (8, 9). The first study to demonstrate the importance of CBG used contrasted pig breeds, the Chinese Meishan vs. the European Large White breed (10). The former shows plasma cortisol concentrations higher than most European pig breeds, as well as a reduced growth rate, low muscle content, and high-fat deposits, all features that may be consequences of their high cortisol levels (11). This genetic mapping study resulted in the detection of a locus containing the gene encoding CBG (called SerpinA6), strongly associated with cortisol levels, in particular stress levels, and explaining 20% of the variance in the F2 population (12). Because CBG was posited as a serious candidate gene of cortisol variability, it was thus investigated further in a follow-up study. New arguments in favor of its involvement were provided by a genetic mapping analysis on the same F2 pig intercross using CBG levels as a trait instead of cortisol. This genetic mapping detected exactly the same genomic locus than when using cortisol levels with higher statistical strength. In addition, CBG mRNA and binding capacity were found different between the parental pig breeds, i.e., higher levels in the Meishan breed (13).

Few years later, a similar genetic mapping approach was used in rodent models. The genome scan detected a highly significant quantitative trait locus associated with poststress corticosterone at the SerpinA6/CBG locus. The cDNA sequence analysis of parental strains revealed a Met276Ile mutation in WKY rat identical to that found in the Biobreeding rat strain that exhibits decreased CBG binding affinity for corticosterone (14). More recently, a series of studies conducted in different pig lines confirmed the genetic linkage or genetic association between SerpinA6/CBG locus and basal cortisol levels in pig (15–18). Finally, in humans, a very significant association between the SERPINA6/SERPINA1 locus and morning plasma cortisol concentrations was reported in a genome-wide association meta-analysis using ~2.5 M SNPs in 12,597 Caucasian subjects, replicated in 2,795 participants (19). Further investigation using part of the cohort showed that common SNPs in this locus were associated with variation in plasma CBG concentrations. These data were replicated in a recent candidate gene study, where SNPs within the SERPINA6 gene were also found associated with morning cortisol and CBG concentrations in a cohort of 1,077 adolescents (20).

In conclusion, CBG appears to be a major component of GC genetic variability in various mammalian species including humans. Given the role of GC in energy metabolism, the association of the locus CBG with body composition and metabolic parameters has also been investigated.

## Genetic Studies Relating CBG and Body Composition

In the first pig study, genetic linkage between the CBG locus and several parameters of body composition was found, namely, backfat weight and thickness, as well as muscle content (13). Interestingly, in this study, plasma CBG levels appeared to be a better predictor of body composition than cortisol levels, as CBG binding capacity showed negative correlation to muscle and positive ones to fat deposits, whereas no significant correlation was found between the same traits and cortisol concentrations. Later, a correlation between the percentage of fat and CBG binding capacity was replicated in a Meishan × Large White F10 intercross (21). However, no genetic linkage to body composition traits was detected in another pig study involving a different breed (Duroc instead of Meishan) (15).

In rodents, many genetic studies have detected adiposity, body weight, insulin levels, or diabetes susceptibility to the locus containing CBG without necessarily testing or even invoking CBG as the causal gene (7).

In humans, a genetic analysis was conducted in a cohort of 44 obese premenopausal women using a genetic marker within the CBG gene (22). No association was found with metabolic or obesity parameters and the CBG genetic marker. However, in patients holding a specific allele of the CBG marker (allele 90), a strong correlation was found between salivary cortisol after dexamethasone suppression test and waist-to-hip ratio, whereas this correlation was not significant for the other patients of the cohort. These results suggest that CBG gene polymorphisms may modulate the influence of the HPA axis on the fat mass distribution in this sample of obese women. In a second study, the same genetic marker in CBG gene was used to genotype a population of 295 men with body mass index (BMI) ranging from 19 to 55 kg/m^2^ (23). The frequency of the allele 90 was found markedly increased among men with morbid obesity (BMI > 40) compared to the rest of the population. Furthermore, the CBG polymorphism was found correlated with BMI and waist circumference in the total population. Finally, this CBG gene polymorphism was also used in a cohort of 45 prepubertal obese children. No differences were found for obesity or metabolic parameters between genotypes at the CBG locus, although allele 90 carriers presented increased 24-h free urinary cortisol (24).

Overall, the genetic studies revealed a modest influence of CBG on body composition and obesity. This modest influence of CBG may be explained by the complexity of the regulation of these traits as well as the small size of the populations studied.

## Dissecting Metabolic Alterations in CBG-Deficient Mouse Models

Older studies have reported altered levels of CBG in animal models of obesity. For example, the obese *Zucker* rat presents plasma and adipose tissue CBG levels twice lower than its lean control. Furthermore, in the obese *Zucker* rat, the regulation of CBG by GCs is lost (25, 26). However, in the *Obese* strain chicken model, contrary to the obese *Zucher* rat, CBG is found twice as high as compared with its healthy control (27). The availability of CBG-deficient mice produced by specific knockout of the SerpinA6 gene provided the opportunity to clarify the specific role of CBG in obesity.

A mouse model of CBG deficiency has been first reported in Petersen et al. (28). When submitted to a 6-week-long high-fat diet (HFD: 26 vs. 4% kcal fat for control diet), Cbg KO showed a tendency (*p* = 0.08) for increased body weight compared to WT. However, the weight loss, as well as liver gene expression estimated by gene arrays, was found equivalent between genotypes after 36 h of food deprivation. Using the same mouse model, a recent study examined the metabolic consequences of a 12-week-long very HFD (60 vs. 18% kcal fat for control diet) on body weight, white adipose tissue, and liver (29). Under the very HFD, total body weight gain and food intake were similar in both Cbg KO and WT groups. Interestingly, Cbg KO mice presented a significantly lower subcutaneous white adipose tissue accumulation than WT mice, associated with a slight higher increase in retroperitoneal and epididymal fat, but no differences in mesenteric fat depots. Adipocytes size increased with HFD in both WT and Cbg KO. However, in agreement with the depot-specific fat weights, the increase in adipocytes’ size was smaller in the subcutaneous fat from Cbg KO than WT, but bigger in the epididymal fat. Total lipid content in liver of animals under HFD was equivalent between genotypes, but the accumulation of lipids in liver under HFD compared to control diet was found higher in Cbg KO compared to WT. HFD similarly increased serum glucose, insulin, leptin, and cholesterol in both genotypes. Non-esterified fatty acids, triacylglycerols, and urea differed neither between diets nor between genotypes.

In both studies, total corticosterone levels were much lower, whereas free corticosterone levels were higher in Cbg KO compared to WT under both control and HFD in blood sampled in the morning, i.e., at the nadir of secretion in mice. CBG and total corticosterone levels were increased by the 60% HFD in WT, but free corticosterone was not changed as CBG was also increased. Cbg KO displayed a modest increase in total and free corticosterone under HFD. In both studies, overall no differences in liver mRNA expression were detected between genotypes under any of the diets, even for GC target genes, such as phosphoenolpyruvate carboxykinase (PEPCK), tyrosine aminotransferase (TAT), or glucose 6-phosphatase (G6PT), in contradiction with the finding of increased free corticosterone in Cbg KO. In the study by Gulfo et al., a global genotype effect was found for liver 11-beta hydroxysteroid dehydrogenase type 2 (11HSD2), which showed reduced expression in Cbg KO mice and a genotype by diet interaction detected for hexose-6-phosphate dehydrogenase (H6PDH) which expression increased under HFD in Cbg KO contrary to WT. Within fat depots, an opposite pattern of expression was found in epididymal fat, i.e., 11HSD2 was increased in Cbg KO as compared to WT, both under control diet and HFD, while 11HSD2 mRNA was very low in subcutaneous fat in both genotypes regardless of the diet. The authors discuss these results as a within-tissue mechanism to regulate GC excess, based on the finding that 11HSD1 and 11HSD2 strongly regulate local availability of GC. In liver, low 11HSD2 expression would normalize GC signaling by reducing the availability of 11-dehydrocorticosterone that is a substrate of 11HSD1 for regeneration of corticosterone. In epididymal fat, increased 11HSD2 mRNA would inactivate GC, by metabolizing corticosterone in 11-dehydrocorticosterone, as a way to counteract the enlargement of adipocyte area. These interpretations may explain the lack of variation between genotypes in mRNA expression of GC target genes in some tissue, despite higher free GC levels in Cbg KO. However, data collected by our group in Bordeaux provide another line of discussion on these data.

We have also generated a Cbg KO mouse model (30) by first constructing a mouse line floxed for the gene Cbg and then crossing these mice with CMV-Cre mice. In our studies, total and free corticosterone levels were measured not only in the morning (9:00 a.m.) but also in the evening (9:00 p.m.) when the secretion of GC is at its peak (mice are nocturnal animals). In the morning, we found increased free GC levels in Cbg KO as in the study by Gulfo et al. (29) or Petersen et al. (28), but in the evening, this genotype difference was no longer present because the higher total GC levels in WT compensated the increased free GC percentage in Cbg KO. Thus, the lack of genotype effect on mRNA expression in basal conditions is expected since when free GC are high (at night), there are no genotype differences in their levels.

There is a discrepancy between our reports and those of Petersen et al. (28) and Gulfo et al. (29), on the levels of free GC measured after stress, when the rise in GC is important. The latter found either no significant differences in free GCs after stress between WT and Cbg KO or increased levels for Cbg KO, whereas we have shown that Cbg KO show a hypoglucocorticoid response to stress compared to WT (31, 32), including after chronic stress (33). Furthermore, Petersen et al. (28) found a phenotype of hyporesponsiveness to GC after septic shock in the Cbg KO mice. To explain this paradox, they hypothesized that Cbg KO mice have become insensitive to high levels of free GC, but they did not provide data on the mechanism involved. On their side, Gulfo et al. (29) found higher free GC levels in Cbg KO mice, both under normal and very HFD, associated with lower thymus and higher adrenals weight, thereby pleading in favor of chronically elevated free GC levels. Taken together, the data from the different groups are confusing regarding whether Cbg KO mice are a model of hyper- or hypocorticosteronism. We believe that methodological differences are another point to take in account. We have used isotopic dilution to precisely evaluate free GC, while the other groups have measured free GC directly in the serum samples for levels that are at the limit of ELISA detection. In their studies, free GC levels are lower than expected in WT mice (0.7 and 1% of the total corticosterone, respectively, instead of 7–10% in our studies). This conflicting data will be important to clarify to interpret the data obtained on metabolic alteration in Cbg KO mice.

## Unraveling the Role of CBG in Human Metabolic Disorders and Obesity

In humans, plasma CBG was found to be a marker of insulin secretion, being negatively or positively correlated with various markers of obesity (BMI and waist-to-hip ratio) depending on the degree of insulin resistance (34, 35). Indeed, insulin was shown to downregulate CBG levels *in vitro* (36), thereby providing an explanation of low CBG levels in obese hyperinsulinemic subjects and high levels of CBG in diabetic insulin-resistant patients. Plasma levels of CBG were also found to correlate with some markers of the metabolic syndrome (fasting glucose, insulin resistance, and adiponectin) and inflammation (interleukin-6 and C reactive protein) (37–39).

By virtue of its SERPIN structure, CBG is cleaved by the serine protease neutrophil elastase, thereby reducing markedly GC binding and enabling massive local delivery of GCs at the site of inflammation (40). Because elevated levels of elastase have been associated with obesity and insulin resistance (41), a recent study examined the respective levels of high- and low-affinity CBG forms, in a cohort of 100 volunteers. Surprisingly, CBG cleavage was found to be reduced in subjects with central obesity and metabolic syndrome, which was interpreted as the sign of altered CBG protein in obesity that would no longer be sensitive to elastase cleavage. The mechanism of reduced CBG cleavage in obese patients remains to be described (42).

## Conclusion

From the literature, it is now well established that CBG is a major factor influencing GCs levels and thus GCs action in tissues. The genetic studies provided hints that CBG was associated with increased fat deposits. Few studies have investigated the direct effects of CBG on energy metabolism and obesity. In a model of CBG deficiency in male mice, CBG seems to play a role in lipid metabolism and storage associated with changes in enzymes controlling local GC availability when animals are submitted to a 60% HFD. A potential effect on appetite is not reported, but this function has been studied only grossly. For example, examination of the pattern of eating through the day and the night or nutrient preferences may reveal a role for CBG. In addition, the status of GC activity in Cbg KO mice is contradictory in the literature, precluding clear-cut interpretation of the effect of CBG deficiency on GC-driven metabolism.

Overall, a role of CBG in GC-driven metabolic disorders is emerging but in extreme conditions of very hyperlipidic diets. The role of elastase in obesity and its regulation of CBG binding affinity have opened new avenues of research. Another area not explored yet is the influence of changes in temperature on CBG binding in the context of obesity. The liver being a very metabolic organ with a higher temperature than surrounding tissues, CBG affinity for GCs is probably diminished there. The influence of CBG in metabolic diseases is probably subtle but could be promising as a therapeutic target, given that CBG is easily accessible in the blood.

## Author Contributions

All the authors listed have made substantial, direct, and intellectual contribution to the work and approved it for publication.

## Conflict of Interest Statement

The authors declare that the research was conducted in the absence of any commercial or financial relationships that could be construed as a potential conflict of interest.
